# Healthcare cost comparison analysis of nivolumab in combination with ipilimumab versus nivolumab monotherapy and ipilimumab monotherapy in advanced melanoma

**DOI:** 10.1186/s40164-019-0138-9

**Published:** 2019-07-03

**Authors:** Ravi Potluri, Sandip Ranjan, Hitesh Bhandari, Helen Johnson, Andriy Moshyk, Srividya Kotapati

**Affiliations:** 1 SmartAnalyst Inc., New York, NY USA; 2 SmartAnalyst India Pvt. Ltd., Gurgaon, India; 3 Bristol-Myers Squibb, Uxbridge, UK; 4 Bristol-Myers Squibb, Lawrence Township, NJ USA

**Keywords:** Advanced melanoma, Melanoma, Nivolumab, Ipilimumab, Healthcare cost comparison, CheckMate 067, NCT01844505

## Abstract

**Background:**

Monoclonal antibodies targeting the cytotoxic T-lymphocyte-associated antigen 4 (CTLA4) (e.g., ipilimumab [IPI]) and the programmed cell death-1 (PD1) receptor (e.g., nivolumab [NIVO]) represent significant breakthroughs in the treatment of advanced melanoma. A combination of the 2 agents has demonstrated efficacy and survival benefits over NIVO or IPI monotherapy in treating advanced melanoma. We compared melanoma-specific costs following treatment with NIVO + IPI, NIVO monotherapy, or IPI monotherapy from the UK and German perspectives to ascertain whether these clinical benefits resulted in a cost advantage.

**Methods:**

Patient-level resource utilization data for the three treatment cohorts were obtained from the CheckMate 067 trial (NCT01844505). All melanoma-specific resources, including drugs (index, concomitant and subsequent melanoma medications), office visits, emergency room visits, hospitalizations, lab tests, procedures and surgeries, utilized over a 48-month evaluation period after start of index treatment were included. Unit costs specific to each geography were applied from external sources. Mean costs per surviving patients were calculated for each successive 30-day period from treatment start and aggregated over the evaluation period.

**Results:**

The total per-patient costs incurred by advanced melanoma patients over the 48-month period following treatment initiation with NIVO + IPI were 9% lower than NIVO monotherapy (£226k vs £248k) and 3% lower compared to IPI monotherapy (£226k vs. £233k) in the UK. In Germany, the total costs incurred by NIVO + IPI cohort were 5% lower than NIVO monotherapy (€258k vs €271k) and 4% lower compared to IPI monotherapy (€258k vs. €268k). Drug costs accounted for > 85% of total costs. Non-drug costs were slightly higher for NIVO + IPI and IPI monotherapy because of higher hospitalization rates. Costs incurred on subsequent drugs post progression were about 45% and 65% lower in NIVO + IPI cohort compared with NIVO and IPI monotherapy cohorts respectively.

**Conclusions:**

The total costs incurred by a patient over a 48-month period following treatment initiation with NIVO + IPI are lower when compared with patients initiating monotherapies; further, the cost advantage is seen to be increasing over time. The clinical benefits offered by the regimen are thus supplemented by a cost advantage, as patients receiving either monotherapy treatment experience faster progression and, consequently, higher subsequent treatment costs. (Note: The cost results reported here are specific to the UK, and Germany, and may not be generalizable to other geographies).

**Electronic supplementary material:**

The online version of this article (10.1186/s40164-019-0138-9) contains supplementary material, which is available to authorized users.

## Background

Cutaneous malignant melanoma is an aggressive cancer type with a rising global incidence over the past 50 years, especially in Europe [1]. In Europe, melanoma accounted for an estimated 100,300 cases and 22,200 deaths in 2012 [2]. In the United States, incidence rates doubled between 1982 and 2011, and are projected to reach 112,000 new cases annually by 2030 in the absence of new interventions [3].

Melanoma that has progressed beyond stage 2 is no longer localized, being classified as regional (stage 3) or distant metastatic melanoma (stage 4) [4]. Treatment of stage 4 melanoma is particularly challenging, with 5-year survival rates (between the years 2005–2011) only 17% as compared to 98% for localized melanoma patients, due in part to the lack of effective treatments for advanced melanoma [5].

Targeted agents such as the selective BRAF and MEK inhibitors and their combinations have improved progression-free survival (PFS) and overall survival (OS) in melanoma patients harbouring the V600 mutation but are effective in only 40% of patients and are associated with resistance that develops within 6 months of initiation of therapy [6–10].

Approvals of novel immunotherapeutic agents such as the cytotoxic T-lymphocyte-associated antigen 4 (CTLA4) inhibitor ipilimumab (IPI) and the anti-programmed cell death-1 (PD1) receptor antibodies pembrolizumab and nivolumab (NIVO) have been regarded as significant breakthroughs in the treatment of advanced melanoma [11–14]. Given the multiple mechanisms through which tumors can evade immune responses, it was postulated that co-administration of 2 non-redundant checkpoint inhibitors may enhance efficacy over monotherapy while having a manageable safety profile. This has been demonstrated in the form of superior response rates, significantly higher PFS, and acceptable tolerability with the combination of NIVO and IPI compared with either agent alone [15, 16].

The clinical efficacy of NIVO in combination with IPI in advanced melanoma patients, with and without the BRAF V600 mutation, has been demonstrated in the CheckMate 067 study [15, 17–19]. Median PFS was 11.5 months (95% confidence interval [CI] 8.7 to 19.3) with NIVO + IPI vs. 6.9 months (95% CI 5.1 to 10.2) with NIVO monotherapy and 2.9 months (95% CI 2.8 to 3.2) with IPI monotherapy [19]. Rates of OS at 3 years among patients in the NIVO + IPI, NIVO monotherapy, and IPI monotherapy arms were 53%, 46%, and 30%, respectively [19]. Based on the clinical efficacy demonstrated in this and other studies, NIVO was approved as a monotherapy and in combination with IPI for the treatment of advanced melanoma in adults by the Food and Drug Administration (FDA) in the United States and the European Medicines Agency (EMA) in Europe.

It is important to determine how the relative effectiveness of these competing treatment options are reflected in the use of resources and costs over the course of a patient’s disease trajectory. In particular, it would be of interest to assess whether the superior efficacy demonstrated by the NIVO + IPI regimen compared to NIVO and IPI monotherapies translates into healthcare cost advantages in the subsequent lines of treatment. This study was carried out to evaluate and compare the melanoma-specific healthcare costs for advanced melanoma patients initiating treatment with the NIVO + IPI combination as well as NIVO and IPI alone over a period of 48 months from the initiation of the respective treatments from the UK and German cost perspectives.

## Methods

### Resource utilization

Individual patient-level data from the ongoing CheckMate 067 clinical trial (NCT01844505) [15, 17, 18] were used to obtain the resource utilization data for the analysis. Briefly, CheckMate 067 is a double-blind, phase 3 trial wherein patients were assigned in a 1:1:1 ratio to receive one of three regimens: NIVO + IPI (NIVO [1 mg/kg of body weight] every 3 weeks plus IPI [3 mg/kg] every 3 weeks for four doses, followed by NIVO [3 mg/kg] every 2 weeks), NIVO alone (NIVO [3 mg/kg] every 2 weeks plus IPI-matched placebo), or IPI alone (IPI [3 mg/kg] every 3 weeks for 4 doses plus NIVO-matched placebo). Treatment was continued until disease progression, development of unacceptable toxic events, or withdrawal of consent. Patients experiencing clinical benefit but not substantial adverse events could be treated beyond progression according to the investigator’s discretion.

For the UK and German payer perspectives, only patients from EU countries were included in the analysis (n = 177, 170, and 167, respectively, for the NIVO + IPI, NIVO monotherapy, and IPI monotherapy cohorts, for a total of 514 patients). Data for each patient were consolidated for each successive month from the start of treatment until the earlier of end of follow-up or 48 months.

All melanoma-specific resources utilized from the day of initiation of treatment with study drugs, including those used prior to progression and after progression, were included in the analysis. Drug resources were further classified as index drugs (NIVO and IPI), subsequent melanoma drugs (melanoma drugs used after discontinuation of treatment with index drugs), and concomitant drugs (pre-medication with index drugs, other drugs used for management of disease or drug-related toxicities). Non-drug resource categories included in the analysis were hospitalizations, surgeries, procedures, laboratory tests, and consultations. Within each drug and non-drug resource category, the most frequently used resources (representing > 90% of the resources within each cost category) were accounted for individually (see Additional file 1: Table S1, which provides the basis for inclusion of the resources), while all other resource subtypes, if any, within the category were aggregated into a single resource subtype designated ‘Others’ and included in the analysis.

The number of vials of the index medications (NIVO and IPI) used were estimated based on the actual drug used by the patient, derived by multiplying the recorded dose level and patient weight captured in the trial data. Vial sharing was not assumed. Dosing information was however not available in the data for the non-index medications (subsequent melanoma and concomitant drugs); the numbers of vials/units used were computed based on dosage and dosing schedules from the drug’s label and published literature and applied for the duration of treatment recorded in the data.

In determining hospitalization resource use, the length of inpatient stays of a patient were considered. For the other non-drug resource categories, counts of all unique instances of resource use were assessed. Non-drug resources were classified as outpatient or inpatient resources, depending upon whether the date of use corresponded to a hospital stay.

Identification of melanoma-specific resources was done on the following basis: (i) medications if having a tag of “cancer”; (ii) concomitant medications if having a tag of “pre-medication” or “adverse events”; (iii) procedures if having a tag of “adverse events”; and (iv) for all other resource categories (hospitalizations, laboratory tests, surgeries, and consultations), all resources used were considered to be melanoma-specific, as these resource records did not have any tag.

All melanoma-specific resources used under each resource type were aggregated for each 30-day period from the start of a patient’s treatment with the index drugs to estimate the month-on-month quantity of resources used by that patient.

### Unit costs

Cost data were not available in the CheckMate 067 trial. As a result, unit costs for each relevant resource were compiled from external, published sources and applied to the resource use obtained from the trial data.

#### UK analysis

For the UK analysis, the unit costs for drugs were obtained from the British National Formulary (BNF) 74th edition (September 2017–March 2018, electronic Market Information Tool (eMIT 2017), and the Monthly Index of Medical Specialties (MIMS).

Unit drug administration costs and all non-drug resource types were obtained from NHS Reference costs (2016–2017). Unit drug administration costs for the index and subsequent melanoma drugs were taken based on the relevant currency codes for delivery of chemotherapy, the selection of which was based on the maximum infusion time recommended in the label or secondary literature [20].

The costs were then adjusted to 2018 prices using the Personal Social Services Research Unit (PSSRU) inflation indices [21].

#### German analysis

For the German analysis, unit costs for drugs were based on payers’ prices estimated from Lauer Taxe as pharmacy selling prices (AVP) for the respective drugs minus the governmental rebate (between 0% and 16%) and pharmacists’ discounts (most commonly €1.77 per pack).

Unit drug administration costs for the index medications and other melanoma drugs were obtained from EBM 2017. Unit costs for the most-used non-drug resources were obtained from EBM 2017 and G-DRG 2017. The costs were then adjusted to 2018 prices using the the health care component of the consumer price index [22].

Individual unit costs were sourced, as described above, for > 90% of the most frequently utilized resources within each cost category (see Additional file 1: Table S1, which provides the basis for inclusion of the resources). For the remaining resources, unit costs were uniformly taken to be the average of unit costs of all resources in that category where specific unit costs were compiled, excluding the costliest 5% of the resources in that category. Unit cost of key resources are available in Additional file 2: Table S2.

For subsequent melanoma drugs where unit costs were not available from the respective local sources, the following assumptions were made: (i) for investigational drugs, unit costs were estimated by benchmarking the monthly costs to the average monthly costs for following melanoma therapies: nivolumab, pembrolizumab, dabrafenib, trametinib, vemurafenib, and cobimetinib, and (ii) unit costs for fotemustine, carmustine, melphalan (Germany analysis) and treosulfan (Germany analysis) were estimated as the weighted average cost of other chemotherapies for which costs were available from respective sources.

### Computation of costs

Month-on-month costs for individual patients were computed for each resource type by multiplying month-on-month quantity of resources used with the respective unit costs. For calculation of index drug use, the number of vials used for the drug in each month, as reported in CheckMate 067 trial data, were multiplied with the respective cost per vial as showing in the table contained in Additional file 2: Table S2, to arrive at the month cost relating to use of the drug. These costs were then grouped into three cost classes—drug, inpatient, and outpatient.

Censoring of patients at different time points was accounted for by censor-adjusting the month-on-month costs to obtain the average per person month-on-month costs for each cost class as follows: (i) The cost for censored patients in a particular month was estimated on the basis of the cost that these patients had incurred historically during the period they were still not censored relative to the current patients at risk during the same period, and (ii) this ratio was then applied to the average cost of the patients at risk during the month under consideration to derive the adjusted cost for the patient cohort. For example, if patient *#j* was censored in month *i* and the average cumulative costs of treatment incurred by patient *#j* and all the patients in the cohort until month (*i *− *1*) are *c*_*i*−*1*_ and C_i−1_, respectively, the ratio (R_ji_) of the cumulative cost until month (*i *− *1*) between patient *j* (before censoring) and the pool of patients at risk equals *c*_*i*−*1*_/C_i−1_. The average of the ratios (R_ji_) across all patients is multiplied by the monthly average cost for patient (estimated by dividing the total costs in the month by the number of patients at risk) in any month *i* to obtain the censor-adjusted per-patient costs for month *i*.

### Comparison of costs between the treatment cohorts

The resultant month-on-month per-patient costs for all three cohorts (NIVO + IPI, NIVO monotherapy, and IPI monotherapy) were compared over the first 48 months from initiation of treatment under the following heads: (i) Overall, at an aggregate level, (ii) in 12-monthly time periods (1–12 months, 13–24 months, 25–36 months, and 37–48 months), (iii) cost classes—drug, inpatient, and outpatient, and (iv) pre-progression index drugs, pre-progression concomitant drugs, post-progression melanoma drugs including the continued use of index drugs after progression, and post-progression concomitant drugs.

## Results

The mean follow-up periods were 30.0 months, 21.7 months and 28.8 months for patients that initiated treatment with NIVO + IPI, IPI monotherapy, and NIVO monotherapy respectively (Table 1). Over the period of 48 months from treatment initiation, mean durations of treatment with the index regimen for the respective cohorts were 10.8 months, 1.8 months and 14.2 months (Table 1).

**Table 1:** Follow-up and duration of treatment (over 48 months from treatment initiation)

	NIVO (n = 170)	NIVO + IPI (n = 177)	IPI (n = 167)
Follow-up (months)			
Mean	28.8	30.0	21.7
Median	35.6	32.2	15.0
Duration of treatment (months)^a^			
Mean	14.2	10.8	1.8
Median	5.5	2.5	2.1

*NIVO + IPI* nivolumab + ipilimumab cohort, *NIVO* nivolumab monotherapy cohort, *IPI* ipilimumab monotherapy cohort

^a^Duration of treatment has been taken to be the period from treatment start to last day of use of a drug

**Fig. 1:**
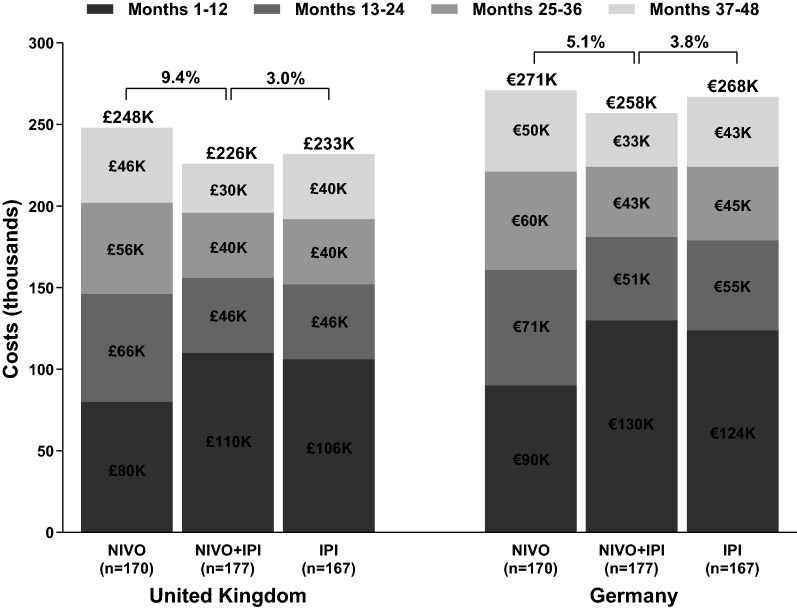
Total melanoma-specific costs (48 months) split across successive time intervals, per patient. *NIVO + IPI* nivolumab + ipilimumab cohort, *NIVO* nivolumab monotherapy cohort, *IPI* ipilimumab monotherapy cohort

The total per-patient melanoma-specific healthcare costs incurred over the 48-month period from initiation of treatment were ₤226k, ₤233k, and ₤248k in the UK, and €258k, €268k, and €271k in Germany for the NIVO + IPI, IPI monotherapy, and NIVO monotherapy cohorts, respectively (Fig. 1). Drug costs (index drugs, concomitant medications, and subsequent treatments) accounted for > 85% of the total healthcare costs.

Despite higher treatment costs in the initial 12 months arising from treatment with a combination of drugs (NIVO and IPI), the total costs incurred by the NIVO + IPI cohort are lower than those incurred by the NIVO monotherapy cohort over a period of 48 months (Fig. 1; lower by 9%, and 5% in the UK, and Germany, respectively). This is attributed to (A) delayed progression among NIVO + IPI patients despite significantly higher treatment discontinuation (40% vs. 13% for the NIVO cohort) [19] and (B) a lower proportion of patients in the NIVO + IPI cohort initiating a subsequent therapy (36% vs. 50% for NIVO monotherapy), resulting in lower post-progression costs associated with the regimen (Table 2). The break-out of total cost into pre- and post-progression costs in each 12-month period has been provided in Additional file 3: Table S3.

**Table 2:** Split of drug costs into pre- and post-progression periods, per patient

	Pre-progression index drugs	Pre-progression concomitant drugs	Post-progression index drugs	Post-progression subsequent melanoma drugs	Post-progression concomitant drugs	Pre-progression drug costs	Post-progression drug costs	Total drug costs
UK analysis								
NIVO + IPI (n = 177)	£135,584	£4994	£17,021	£54,096	£3262	£140,578	£74,379	£214,957
IPI (n = 167)	£66,115	£1081	£4413	£145,037	£4032	£67,196	£153,482	£220,678
NIVO (n = 170)	£109,653	£1621	£25,936	£99,448	£2843	£111,274	£128,266	£239,540
Difference (NIVO + IPI vs. IPI	105%	362%	286%	− 63%	− 19%	109%	− 52%	− 3%
Difference (NIVO + IPI vs. NIVO)	24%	208%	− 34%	− 46%	15%	26%	− 42%	− 10%
Germany analysis								
NIVO + IPI (n = 177)	€133,743	€10,621	€17,106	€52,940	€8897	€144,364	€78,943	€223,307
IPI (n = 167)	€63,773	€2646	€4250	€152,395	€9865	€66,419	€166,510	€232,929
NIVO (n = 170)	€110,977	€5901	€26,235	€97,889	€7749	€116,878	€131,873	€248,751
Difference (NIVO + IPI vs. IPI	110%	301%	302%	− 65%	− 10%	117%	− 53%	− 4%
Difference (NIVO + IPI vs. NIVO)	21%	80%	− 35%	− 46%	15%	24%	− 40%	− 10%

*NIVO + IPI* nivolumab + ipilimumab cohort, *NIVO* nivolumab monotherapy cohort, *IPI* ipilimumab monotherapy cohort

When compared to the total costs incurred over the 48-month period by the IPI monotherapy cohort, the NIVO + IPI cohort incurs slightly lower costs in the UK and Germany (by 3% in the UK and by 4% in Germany). Unlike in the comparison with the NIVO monotherapy cohort where the NIVO + IPI cohort had substantially higher costs in the initial 12 months but markedly lower costs thereafter, the comparison with the IPI monotherapy cohort shows comparable costs consistently through the evaluation period, driven by the significant upfront costs of IPI therapy in both the cohorts (Fig. 1). Costs incurred by the combination regimen cohort were 4%, 3%, and 2% higher than those incurred by the IPI monotherapy cohort during the first 12, 24, and 36 months, being 3% lower at 48 months. In Germany, the corresponding costs were 5%, 1%, and 0.3% higher at 12, 24, and 36 months, and almost 4% lower at the 48-month mark. Similar to the NIVO monotherapy cohort, patients in the IPI cohort experience faster progression, resulting in a substantially higher proportion of patients initiating subsequent melanoma medications (65% vs 36% for NIVO + IPI cohort).

**Fig. 2:**
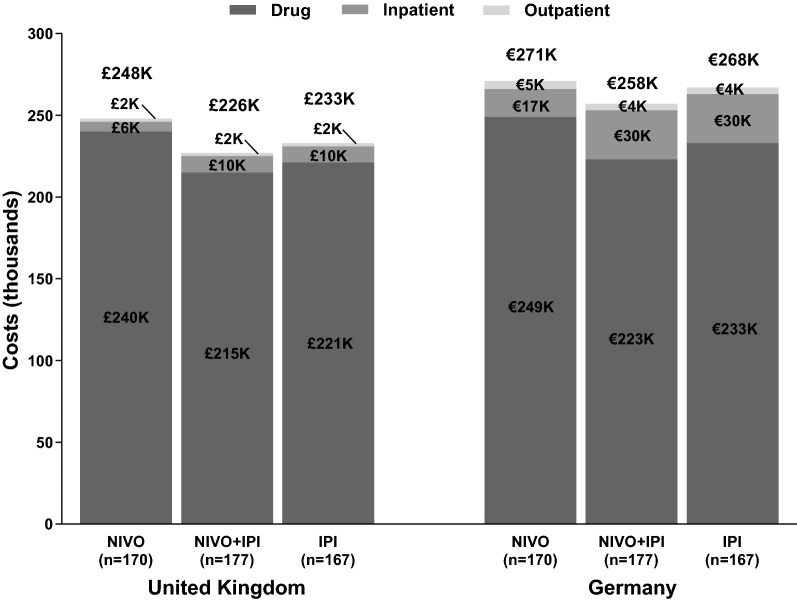
Total melanoma-specific costs (48 months) split into three cost categories, per patient. *NIVO + IPI* nivolumab + ipilimumab cohort, *NIVO* nivolumab monotherapy cohort, *IPI* ipilimumab monotherapy cohort

Drug costs (including index drugs, concomitant medications and subsequent treatments) accounted for > 85% of all melanoma-specific costs consistently across all three cohorts over the first 48 months (Fig. 2). Disaggregating the drug costs for the cohorts into the pre-progression and post-progression periods revealed that pre-progression drug costs were higher for the NIVO + IPI cohort by 26% and 109% over the NIVO monotherapy and IPI monotherapy cohorts respectively, while post-progression drug costs with the NIVO + IPI combination regimen were 42% and 52% lower compared with NIVO and IPI monotherapy cohorts respectively in the UK. The corresponding values for Germany are presented in Table 2. Pre-progression concomitant medication costs are higher for NIVO + IPI combination cohort compared to the monotherapy cohorts, reflecting the higher incidence of Grade 3/4 AEs in the NIVO + IPI arm of the CheckMate 067 trial compared to the NIVO monotherapy and IPI monotherapy cohorts (59% for NIVO + IPI, 22% for NIVO monotherapy, and 28% for IPI monotherapy [19]).

Non-drug costs, mainly comprising inpatient costs, are slightly higher for the NIVO + IPI and IPI monotherapy cohorts as compared to NIVO monotherapy cohort owing to the greater hospitalization rate (68% for NIVO + IPI and 64% for IPI monotherapy vs 52% for NIVO monotherapy) and mean days of hospitalization per patient (18 days for NIVO + IPI combination vs 17 days for IPI mono and 11 days for NIVO monotherapy cohorts).

## Discussion

**Fig. 3:**
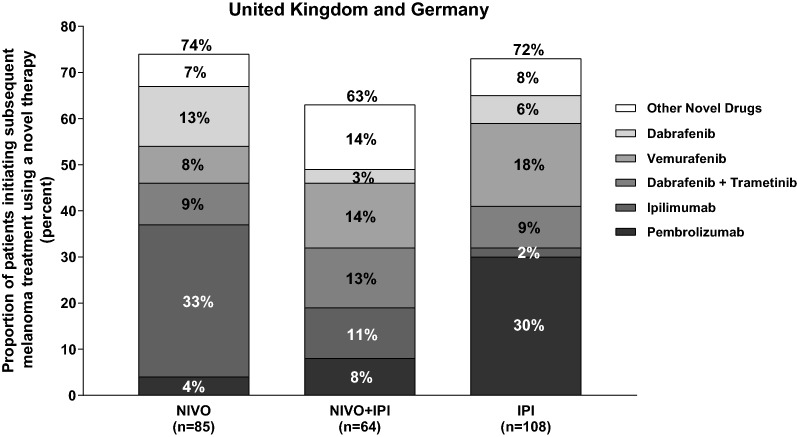
Utilization of novel therapies among patients that initiate subsequent melanoma treatment. n represents the number of patients initiating subsequent melanoma treatment; Of the 177 patients in the NIVO + IPI arm, 64 patients initiate a subsequent melanoma treatment of which 63% patients use one of the novel drugs. *IPI* ipilimumab monotherapy cohort; *NIVO* nivolumab monotherapy cohort; *NIVO + IPI* nivolumab + ipilimumab cohort; *Dab* dabrafenib; *Dab + Tra* dabrafenib + trametinib; *Ipi* ipilimumab; *Other novel drugs* nivolumab, nivolumab + ipilimumab, trametinib, vemurafenib + cobimetinib, binimetinib, encorafenib, binimetinib + encorafenib; *Pem* pembrolizumab; *Vem* vemurafenib

This study determined that melanoma-specific healthcare costs during the first 48 months of treatment for the NIVO + IPI regimen cohort are lower than costs incurred by the NIVO and IPI monotherapy cohorts, despite the higher cost burden imposed on this cohort by index treatment with a combination of drugs. This cost advantage for the NIVO + IPI cohort over the longer term, can be attributed to a combination of factors: (1) the durability of response despite higher treatment discontinuation rates with the combination [23], (2) delayed progression with the combination regimen resulting in a smaller proportion of patients going on to require subsequent melanoma treatment, and (3) lower use of novel melanoma drugs as subsequent therapy (Fig. 3). These findings with respect to the combination regimen are all the more appealing when viewed in conjunction with the superior clinical outcomes associated with it.

**Fig. 4:**
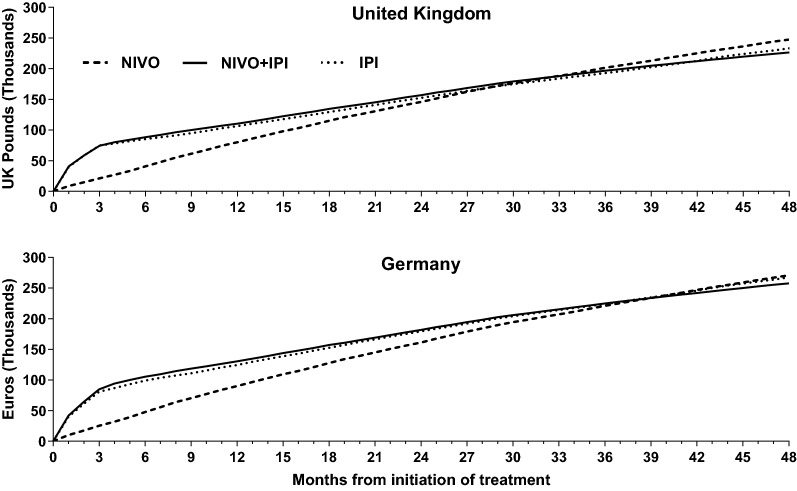
Total melanoma-specific costs, cumulated from initiation of treatment, per patient. *NIVO + IPI* nivolumab + ipilimumab cohort, *NIVO* nivolumab monotherapy cohort, *IPI* ipilimumab monotherapy cohort

The sub-component analyses revealed that costs are higher in the first few months after treatment initiation and gradually decline over the course of time (especially with the NIVO + IPI regimen and with IPI monotherapy). Further, NIVO + IPI cohort’s cost differential with NIVO monotherapy cohort is at its maximum at the end of 1 month from treatment initiation and diminishes continuously over the rest of the 48-month period (Fig. 4). This is consistent with the use of melanoma drugs during the evaluation period, with the early period characterized by the use of a combination of drugs in the NIVO + IPI cohort followed by maintenance and/or treatment-free periods and relatively lower use of subsequent melanoma treatments in the later months. It will be interesting to see whether the cost curves exhibited by the various cohorts and the trends in relative differential between them extend beyond this 48-month follow-up period.

Analyses of pre-progression and post-progression costs in the two geographies revealed that the NIVO + IPI combination regimen is associated with reduced post-progression costs compared with NIVO monotherapy and IPI monotherapy, reflecting and confirming its superior clinical effectiveness in terms of longer PFS and lower proportion of patients initiating a subsequent therapy. Of the three cohorts, the IPI monotherapy cohort has the lowest pre-progression drug costs due to its fixed dosing schedule (maximum of 4 cycles) and early discontinuation of treatment due to toxicity or early progression. It is however noteworthy that the pre-progression costs for the NIVO + IPI combination are relatively subdued due to a higher proportion of patients discontinuing treatment prior to progression (mainly due to toxicity) and not requiring a subsequent melanoma treatment for a longer period [19]. This aspect in fact has been articulated in several publications. Hodi et al. reported that median treatment-free interval was longer in the NIVO + IPI group (15.4 months) compared to the NIVO group (1.7 months) or IPI group (1.9 months). Regan et al. carried out Kaplan–Meier analysis for treatment-free survival and found that 36-month truncated mean TFS was the highest for NIVO + IPI cohort. A post hoc analysis [24, 25] of pooled CheckMate 069 and 067 trials reported an average treatment-free interval of 5.3, 3.4, and 2.3 years with NIVO + IPI, NIVO, and IPI, respectively, extrapolated over a patient’s lifetime. On the other hand, post-progression drug costs are highest for the IPI monotherapy cohort (Table 2), attributed not just to early progression but also to higher utilization of novel therapies such as PD-1 inhibitors, BRAF inhibitors, and MEK inhibitors (Fig. 3).

This cost analysis for the first 48 months after initiation of treatment reveals that NIVO + IPI combination therapy is an economically competitive option for the treatment of newly diagnosed advanced melanoma. It will be of import to evaluate whether the economic advantage associated with the combination regimen is maintained across key melanoma subgroups categorized by BRAF mutation status and PD-L1 expression levels.

Multiple studies have previously carried out health economic analyses in advanced melanoma [26–30]. The results presented in these economic evaluations are not comparable to the results from the current analysis on account of the following differences between the methodologies adopted by the earlier evaluations and our study:

1. All the other studies involve cost-effectiveness analyses (CEA) which takes into account both costs and outcomes and compute the ratio of incremental costs to incremental effectiveness (life years or quality-adjusted life years). The present analysis is solely a cost-evaluation study and does not take into consideration any effectiveness outcomes.
2. The costs reported in these CEAs are based on a set of assumptions around drug and resource use, generally derived from summary data. Our study is based on actual drug and resource utilization as observed in individual patient-level data from CheckMate 067 and hence cannot be compared with what is presented in the available economic studies. In addition, CEA analyses are often conducted over the long term and requires extrapolation beyond the available follow-up period. The present analysis is based on observed data and does not require assumptions on future costs.
3. Owning to the inherent methodology of costs estimation in a cost-effectiveness evaluation, the estimated costs are arrived at after adjusting for survival over the modelled time horizon. The cost results presented in this study have been estimated by aggregating (over the 48-month time period from start of treatment) the average month-on-month cost per surviving patient; this, in essence, reflects the costs incurred by a patient who survives this entire 48-month period.

Our study has a few limitations. Resource utilization data for the cost analysis has been sourced from a Phase III clinical trial and may not adequately reflect real-world resource utilization and treatment patterns. Given that the trial is conducted in controlled settings where participants are expected to be more compliant, drug use may have been overstated in relation to what it would be in routine clinical practice. It is also possible that because of active and regular monitoring, adverse events are detected earlier in clinical trials than they would be in the real-world settings. This analysis was solely a cost evaluation and no health/quality-adjusted life year benefits were considered. The cost results derived from this analysis could be augmented with other pharmacoeconomic evaluations by incorporating long-term projections of outcomes and costs. Due to limited information available from the trial data, all non-melanoma specific resources could not be excluded, implying that the melanoma-specific healthcare costs may actually be lower than the costs reported in this study. The cost results are specific to the UK, and Germany, and may not be generalizable to other geographies.

## Conclusions

The total costs incurred by a patient over a 48-month period following treatment initiation with NIVO + IPI are lower when compared with the monotherapy options; further, this cost advantage is seen to be increasing with time. This demonstrates that not only does the regimen offer clinical superiority over the monotherapies, as seen in CheckMate 067, but that it also proffers a cost advantage, as patients receiving either monotherapy treatment experience faster progression and, consequently, higher subsequent treatment costs. We conclude that it is critical to look beyond the direct prices of novel therapies and combination regimens, and to evaluate costs over a prolonged period of time to realize the potential cost benefits that may result from their superior efficacy and/or safety profiles. It will be important to conduct a similar evaluation based on real-world data when they become available and to assess whether the results seen and conclusions drawn from this analysis of clinical trial data hold true in routine clinical practice. Nevertheless, our findings from this 48-month analysis of clinical trial data provide valuable insights that can help complement evidence on the clinical efficacy and safety of the NIVO + IPI combination regimen and support the case for its adoption as a frontline treatment option in advanced melanoma.

## Additional files

**Additional file 1.** Basis for inclusion of drug and non-drug resources in the cost comparison analysis.

**Additional file 2.** Unit cost of key resources (2017).

**Additional file 3.** Year-wise split of drug costs into pre- and post-progression periods, per patient.

## Data Availability

All data generated or analysed during this study are included in this published article and its additional information files. Questions regarding the datasets may be directed to the corresponding author.

## Abbreviations

AE: adverse event
AVP: Apothekenverkaufspreis (pharmacy retail price of medications in Germany)
BNF: British National Formulary
*BRAF*: gene encoding the BRAF serine-threonine kinase
CTLA4: cytotoxic T-lymphocyte-associated antigen 4
EBM: Einheitlicher Bewertungsmaßstab (Uniform Evaluation Scale in Germany)
EMA: European Medicines Agency
eMIT: electronic Market Information Tool
EU: European Union
FDA: United States Food and Drug Administration
G-DRG: German Diagnosis-Related Group
IPI: ipilimumab
*MEK*: genes encoding the mitogen activated protein kinase enzymes MEK1 and MEK2
MIMS: Monthly Index of Medical Specialities
NIVO: nivolumab
OS: overall survival
PD1: programmed cell death-1
PFS: progression-free survival
PSSRU: Personal Social Services Research Unit
WAC: wholesale acquisition costs
