# Supplementary material for: Healthcare cost comparison analysis of nivolumab in combination with ipilimumab versus nivolumab monotherapy and ipilimumab monotherapy in advanced melanoma
Source: Exp Hematol Oncol. 2019 Jul 3;8:14. doi: 10.1186/s40164-019-0138-9 (PMC6610852; doi:10.1186/s40164-019-0138-9)
Supplement: Supplementary file 1 — Additional file 1. Basis for inclusion of drug and non-drug resources in the cost comparison analysis. [file 40164_2019_138_MOESM1_ESM.docx]

**Table S1.** Basis for inclusion of drug and non-drug resources in the cost comparison analysis

| **Category** | **Retention strategy** | **Count of resource types where respective unit costs were compiled** |
| --- | --- | --- |
| Index drugs | All | 2 |
| Subsequent melanoma drugs | All | 43 |
| Concomitant drugs | Resources adding up to 90% of instances in CheckMate 067 Trial data | 272 |
| Procedures | Resources adding up to 99% of instances (additional qualification, N ≥2) in CheckMate 067 Trial data | 178 |
| Hospitalization | Resources adding up to 99% of instances (additional qualification, N ≥2) in CheckMate 067 Trial data | 73 |
| Laboratory tests | Resources adding up to 99% of instances (additional qualification, N ≥2) in CheckMate 067 Trial data | 45 |
| Surgeries | All resources retained given the high proportion of surgical procedures with low counts | 143 |
| Consultation | All resources retained, given the small list of resource types | 39 |
