# Supplementary material for: Healthcare cost comparison analysis of nivolumab in combination with ipilimumab versus nivolumab monotherapy and ipilimumab monotherapy in advanced melanoma
Source: Exp Hematol Oncol. 2019 Jul 3;8:14. doi: 10.1186/s40164-019-0138-9 (PMC6610852; doi:10.1186/s40164-019-0138-9)
Supplement: Supplementary file 2 — Additional file 2. Unit cost of key resources (2017). [file 40164_2019_138_MOESM2_ESM.docx]

**Table S2.** Unit cost of key resources (2017)

| **Resource Type** | **Unit cost-UK (£)** | **Unit cost-Germany (€)** | **Metric of unit cost** |
| --- | --- | --- | --- |
| Index Drugs |  |  |  |
| Nivolumab | £1,097.0 | €1,246.4 | Cost per vial (10 mg/1 ml, 10 ml solution) |
|  | £439.0 | €504.1 | Cost per vial (10 mg/1 ml, 4 ml solution) |
| Ipilimumab | £15,000.0 | €14,766.2 | Cost per vial (5 mg/1 ml, 40 ml solution) |
|  | £3,750.0 | €3,733.2 | Cost per vial (5 mg/1 ml, 10 ml solution) |
| Subsequent melanoma drugs |  |  |  |
| Dabrafenib | £100.0 | €117.0 | Per dose |
| Ipilimumab | £18,750.0 | €18,499.4 | Per dose |
| Vemurafenib | £125.0 | €100.5 | Per dose |
| Trametinib | £160.0 | €125.8 | Per dose |
| Pembrolizumab | £5,260.0 | €6,177.1 | Per dose |
| Nivolumab (combination use) | £878.0 | €1,008.2 | Per dose |
| Nivolumab | £2,633.0 | €2,997.0 | Per dose |
| Temozolomide | £25.5 | €401.7 | Per dose |
| Cisplatin | £1.1 | €6.6 | Per day |
| Paclitaxel | £2.3 | €78.9 | Per day |
| Carboplatin | £1.3 | €11.2 | Per day |
| Dacarbazine | £7.7 | €19.4 | Per day |
| Binimetinib | £207.8 | €218.1 | Per day |
| Encorafenib | £207.8 | €218.1 | Per day |
| Cobimetinib | £203.6 | €261.3 | Per day |
| Concomitant drugs |  |  |  |
| Acetaminophen | £0.3 | €3.4 | Per day |
| Prednisone | £7.4 | €1.0 | Per day |
| Loperamide | £0.2 | €2.7 | Per day |
| Ibuprofen | £0.2 | €0.6 | Per day |
| Amoxicilin+Clavunate | £0.3 | €5.2 | Per day |
| Dexamethasone | £2.0 | €2.5 | Per day |
| Levothyroxine | £0.1 | €0.5 | Per day |
| Ondansetron | £0.5 | €83.3 | Per day |
| Metoclopramide | £0.4 | €0.9 | Per day |
| Lab tests |  |  |  |
| Kidney function tests | £1.1 | €83.4 | Per lab test |
| Electrolytes | £1.1 | €3.1 | Per lab test |
| Liver function tests | £1.1 | €7.1 | Per lab test |
| Hemogram | £3.1 | €1.5 | Per lab test |
| Lipase | £1.1 | €1.2 | Per lab test |
| Thyroxine, free | £1.7 | €3.7 | Per lab test |
| Amylase | £1.1 | €1.2 | Per lab test |
| Hospitalizations |  |  |  |
| Colitis | £900.8 | €1,606.0 | Per hospitalization day |
| Neuropathy | £580.6 | €2,241.6 | Per hospitalization day |
| Hepatotoxicity | £434.7 | €2,173.2 | Per hospitalization day |
| Pneumonia | £397.6 | €2,658.5 | Per hospitalization day |
| Hepatitis | £434.7 | €2,173.2 | Per hospitalization day |
| Renal failure | £1,270.9 | €3,017.23 | Per hospitalization day |
| Pulmonary embolism | £410.7 | €3,625.5 | Per hospitalization day |
| Diarrhea | £478.7 | €1,858.9 | Per hospitalization day |
| Seizures | £441.8 | €2,149.3 | Per hospitalization day |
| Procedures and surgeries |  |  |  |
| Colonoscopy | £303.0 | €18.4 | Per procedure |
| CT scan | £103.6 | €75.4 | Per procedure |
| Transfusion | £214.2 | €15.8 | Per procedure |
| MRI | £151.2 | €135.1 | Per procedure |
| Echocardiogram | £99.8 | €52.4 | Per procedure |
| Endoscopy | £318.4 | €132.3 | Per procedure |
| Angiogram | £536.1 | €130.1 | Per procedure |
| Culture-blood | £7.7 | €8.8 | Per procedure |
| Electrocardiogram | £137.6 | €12.0 | Per procedure |
| Ultrasound | £69.8 | €7.9 | Per procedure |
| Radiotherapy | £756.1 | €86.0 | Per procedure |
| Biopsy | £262.0 | €40.5 | Per procedure |
| X-ray | £107.2 | €38.7 | Per procedure |
| Resection metastasis brain | £5,952.8 | €10,715.9 | Per surgery |
| Cholecystectomy | £2,408.1 | €3,112.9 | Per surgery |
| Ileostomy | £3,216.0 | €7,561.9 | Per surgery |
| Colectomy | £4,171.0 | €7,561.9 | Per surgery |
| Lymphadenectomy | £772.1 | €2,462.7 | Per surgery |
| Laparotomy | £2,838.2 | €2,462.7 | Per surgery |
| Thoracostomy | £648.0 | €27.3 | Per surgery |
| Appendicectomy | £2,604.6 | €2,860.1 | Per surgery |
| Explorative laparotomy | £2,838.2 | €2,462.7 | Per surgery |
| Exc.-skin | £1,266.4 | €2,462.7 | Per surgery |
| Gamma knife-brain | £2,434.2 | €3,584.5 | Per surgery |
| Consultations |  |  |  |
| GP consult | £172.7 | €17.4 | Per consultation day |
| Hospital outpatient | £172.8 | €20.34 | Per consultation day |
| Emergency room no hospital | £151.0 | €107.5 | Per consultation day |
| Specialist consult | £138.8 | €29.6 | Per consultation day |
| Radiology | £60.9 | €31.0 | Per consultation day |
| Day care | £738.3 | €789.3 | Per consultation day |
| Physiotherapist | £79.6 | €8.3 | Per consultation day |
| Physical examination | £172.7 | €27.8 | Per consultation day |
