# Supplementary material for: Healthcare cost comparison analysis of nivolumab in combination with ipilimumab versus nivolumab monotherapy and ipilimumab monotherapy in advanced melanoma
Source: Exp Hematol Oncol. 2019 Jul 3;8:14. doi: 10.1186/s40164-019-0138-9 (PMC6610852; doi:10.1186/s40164-019-0138-9)
Supplement: Supplementary file 3 — Additional file 3. Year-wise split of drug costs into pre- and post-progression periods, per patient. [file 40164_2019_138_MOESM3_ESM.docx]

**Table S3.** Year-wise split of drug costs into pre- and post-progression periods, per patient

|  | UK | | | | |
| --- | --- | --- | --- | --- | --- |
|  | 1-12 months | 13-24 months | 25-36 months | 37-48 months | 1-48 months |
|  | NIVO+IPI | | | | |
| Pre-progression index drugs | £86,925 | £22,054 | £16,444 | £10,161 | £135,584 |
| Pre-progression concomitant drugs | £1,190 | £1,343 | £1,246 | £1,215 | £4,994 |
| Post-progression index drugs | £5,526 | £5,744 | £3,154 | £2,597 | £17,021 |
| Post-progression subsequent melanoma drugs | £6,464 | £15,492 | £17,555 | £14,585 | £54,096 |
| Post-progression concomitant drugs | £556 | £719 | £957 | £1,030 | £3,262 |
| Pre-progression drug costs | £88,115 | £23,397 | £17,691 | £11,375 | £140,578 |
| Post-progression drug costs | £12,546 | £21,955 | £21,666 | £18,212 | £74,379 |
| Total drug costs | £100,661 | £45,352 | £39,357 | £29,588 | £214,957 |
|  | IPI | | | | |
| Pre-progression index drugs | £66,115 | £0 | £0 | £0 | £66,115 |
| Pre-progression concomitant drugs | £482 | £168 | £218 | £213 | £1,081 |
| Post-progression index drugs | £4,413 | £0 | £0 | £0 | £4,413 |
| Post-progression subsequent melanoma drugs | £25,374 | £42,338 | £38,328 | £38,997 | £145,037 |
| Post-progression concomitant drugs | £927 | £1,242 | £1,286 | £578 | £4,032 |
| Pre-progression drug costs | £66,597 | £168 | £218 | £213 | £67,196 |
| Post-progression drug costs | £30,714 | £43,580 | £39,614 | £39,574 | £153,482 |
| Total drug costs | £97,311 | £43,748 | £39,832 | £39,787 | £220,678 |
|  | NIVO | | | | |
| Pre-progression index drugs | £43,460 | £28,581 | £21,072 | £16,540 | £109,653 |
| Pre-progression concomitant drugs | £288 | £431 | £435 | £467 | £1,621 |
| Post-progression index drugs | £8,524 | £7,281 | £5,086 | £5,044 | £25,936 |
| Post-progression subsequent melanoma drugs | £22,574 | £27,101 | £27,297 | £22,516 | £99,488 |
| Post-progression concomitant drugs | £311 | £655 | £930 | £946 | £2,843 |
| Pre-progression drug costs | £43,748 | £29,013 | £21,507 | £17,007 | £111,274 |
| Post-progression drug costs | £31,409 | £35,038 | £33,313 | £28,506 | £128,266 |
| Total drug costs | £75,157 | £64,050 | £54,821 | £45,513 | £239,540 |

|  | Germany | | | | |
| --- | --- | --- | --- | --- | --- |
|  | 1-12 months | 13-24 months | 25-36 months | 37-48 months | 1-48 months |
|  | NIVO+IPI | | | | |
| Pre-progression index drugs | €84,510 | €22,314 | €16,639 | €10,280 | €133,743 |
| Pre-progression concomitant drugs | €2,856 | €2,528 | €2,712 | €2,525 | €10,621 |
| Post-progression index drugs | €5,475 | €5,811 | €3,192 | €2,628 | €17,106 |
| Post-progression subsequent melanoma drugs | €5,913 | €15,741 | €16,997 | €14,288 | €52,940 |
| Post-progression concomitant drugs | €1,735 | €2,208 | €2,404 | €2,551 | €8,897 |
| Pre-progression drug costs | €87,365 | €24,842 | €19,351 | €12,805 | €144,364 |
| Post-progression drug costs | €13,124 | €23,760 | €22,593 | €19,466 | €78,943 |
| Total drug costs | €100,489 | €48,602 | €41,944 | €32,272 | €223,307 |
|  | IPI | | | | |
| Pre-progression index drugs | €63,773 | €0 | €0 | €0 | €63,773 |
| Pre-progression concomitant drugs | €1,203 | €349 | €480 | €614 | €2,646 |
| Post-progression index drugs | €4,250 | €0 | €0 | €0 | €4,250 |
| Post-progression subsequent melanoma drugs | €25,969 | €45,304 | €40,828 | €40,294 | €152,395 |
| Post-progression concomitant drugs | €2,500 | €3,007 | €2,596 | €1,761 | €9,865 |
| Pre-progression drug costs | €64,976 | €349 | €480 | €614 | €66,419 |
| Post-progression drug costs | €32,720 | €48,310 | €43,424 | €42,056 | €166,510 |
| Total drug costs | €97,696 | €48,659 | €43,904 | €42,670 | €232,929 |
|  | NIVO | | | | |
| Pre-progression index drugs | €44,026 | €28,906 | €21,312 | €16,732 | €110,977 |
| Pre-progression concomitant drugs | €726 | €1,528 | €1,758 | €1,889 | €5,901 |
| Post-progression index drugs | €8,624 | €7,364 | €5,144 | €5,103 | €26,235 |
| Post-progression subsequent melanoma drugs | €21,651 | €26,664 | €27,111 | €22,464 | €97,889 |
| Post-progression concomitant drugs | €1,118 | €1,800 | €2,608 | €2,223 | €7,749 |
| Pre-progression drug costs | €44,752 | €30,435 | €23,070 | €18,621 | €116,878 |
| Post-progression drug costs | €31,393 | €35,828 | €34,863 | €29,790 | €131,873 |
| Total drug costs | €76,144 | €66,262 | €57,933 | €48,411 | €248,751 |
